# Targeting hypoxia in the tumor microenvironment: a potential strategy to improve cancer immunotherapy

**DOI:** 10.1186/s13046-020-01820-7

**Published:** 2021-01-09

**Authors:** Bin Wang, Qin Zhao, Yuyu Zhang, Zijing Liu, Zhuangzhuang Zheng, Shiyu Liu, Lingbin Meng, Ying Xin, Xin Jiang

**Affiliations:** 1 Department of Radiation Oncology, The First Hospital of Jilin University, 71 Xinmin Street, Changchun, 130021 China; 2 Jilin Provincial Key Laboratory of Radiation Oncology & Therapy, The First Hospital of Jilin University, Changchun, 130021 China; 3 NHC Key Laboratory of Radiobiology, School of Public Health, Jilin University, Changchun, 130021 China; 4 Department of Hematology and Medical Oncology, Moffitt Cancer Center, Tampa, FL 33612 USA; 5 Key Laboratory of Pathobiology, Ministry of Education, Jilin University, 126 Xinmin Street, Changchun, 130021 China

**Keywords:** Cancer immunotherapy, Hypoxia, Tumor microenvironment, Hypoxia-inducible factor, Immune suppression, Resistance, Combination approaches

## Abstract

With the success of immune checkpoint inhibitors (ICIs), significant progress has been made in the field of cancer immunotherapy. Despite the long-lasting outcomes in responders, the majority of patients with cancer still do not benefit from this revolutionary therapy. Increasing evidence suggests that one of the major barriers limiting the efficacy of immunotherapy seems to coalesce with the hypoxic tumor microenvironment (TME), which is an intrinsic property of all solid tumors. In addition to its impact on shaping tumor invasion and metastasis, the hypoxic TME plays an essential role in inducing immune suppression and resistance though fostering diverse changes in stromal cell biology. Therefore, targeting hypoxia may provide a means to enhance the efficacy of immunotherapy. In this review, the potential impact of hypoxia within the TME, in terms of key immune cell populations, and the contribution to immune suppression are discussed. In addition, we outline how hypoxia can be manipulated to tailor the immune response and provide a promising combinational therapeutic strategy to improve immunotherapy.

## Background

In the last decade, emerging cancer immunotherapy based on immune checkpoint inhibitors (ICIs) has revolutionized the paradigm of oncotherapy [1]. Monoclonal antibodies against cytotoxic T lymphocyte antigen 4 (CTLA4) or programmed cell death protein 1 (PD-1) and its ligand (PD-L1) have achieved never-before-seen clinical benefit and obtained FDA-approval for more than 15 cancer types [2]. Currently, with more than 3500 ongoing clinical trials investigating the efficacy of immunotherapy (ClinicalTrials.gov), immunotherapy is expected to change the standard of care towards multiple malignancies. Although unsurpassed clinical efficacy has been observed, identification of predictive biomarkers and the development of resistance during or after immunotherapy remain major challenges. The objective response rate of ICI monotherapy varies from 20 to 40% in most tumor types, with a considerable proportion of partial responders [3]. In addition, acquired resistance develops in one-third of patients who initially respond, eventually resulting in disease relapse or progression [3]. With complex resistance mechanisms thwarting the therapeutic outcomes of immunotherapy, there is a pressing clinical need to develop combination approaches so that more patients can benefit from immunotherapy.

It is well known that hypoxia within the tumor microenvironment (TME) is an intrinsic property of all solid malignant tumors [4]. The metabolic profile of the hypoxic TME is characterized by dynamic gradients of oxygen pressure, glycolysis, extracellular acidosis, an accumulation of lactate and adenosine, and depletion of essential nutrients [5]. The hypoxic TME is widely considered an independent prognostic indicator, which is consistently related to poor survival in various cancer types, including head and neck, breast, non-small cell lung, cervical, and ovarian cancers [6]. The hypoxic TME exerts a far-reaching impact on diverse aspects of tumor biology such as tumor cell metabolism, intercellular communication, epitranscriptomics, and epigenomics [7]. Substantial cellular stress caused by the hypoxic TME promotes the heterogeneity and plasticity of tumors and contributes to the development of more invasive and therapeutically resistant tumor phenotypes [4]. Hypoxia in the TME can enhance the resistance of tumor cells to radiotherapy through promoting DNA self-repairing by cells [8]. Hypoxia is also the primary barrier against the therapeutic efficacy of photodynamic therapy (PDT), as the photosensitizers utilize molecular oxygen to transfer near-infrared laser energy to generate high reactive singlet oxygen and other reactive oxygen species (ROS), which directly or indirectly induces tumor cell apoptosis and/or necrosis [9, 10]. With respect to chemotherapy, hypoxia-induced cell cycle arrest, increased nucleophilic substances, elevated DNA repair enzyme activity, decreased ROS, and reduced sensitivity to p53-mediated apoptosis can either directly or indirectly dampen the efficacy of anticancer agents [8, 11]. Moreover, emerging evidence has indicated that hypoxia can cause tumor resistance to immunotherapy by several mechanisms, involving both innate and adaptive immunity. Hypoxia-driven adaptive mechanisms allow tumor cells to continue to survive and even proliferate in the hypoxic TME while also creating an inhospitable environment for immune cells and damaging key regulatory pathways [12]. Hypoxia can induce immune tolerance and immune escape by interfering with tumor killing functions of effector cells and preventing their homing to the TME [13]. Hypoxia can also promote immune-suppressive stromal cell differentiation and increase immunosuppressive factors, including checkpoint molecules, as well as modify the metabolic landscape [12]. Currently available biomarkers to predict immunotherapy efficacy mainly include PD-L1, tumor mutational burden (TMB), and microsatellite instability/deficient mismatch repair (MSI/dMMR); however, the problem of “poor soil” is often ignored [14]. Therefore, hypoxia may be exploited as a potent biomarker to predict immunotherapy outcomes.

Considering the central role of the hypoxic TME in tumor progression and therapeutic resistance, approaches directed towards the hypoxic TME hold considerable promise in innovative immunotherapy guiding. In this review, we discuss the potential impact of hypoxia within the TME, in terms of interference with key immune cell populations, and its contribution to immune suppression, thus providing some suggestions on how to target the hypoxic TME to synergize with cancer immunotherapy.

## Hypoxic tumor microenvironment and hypoxia signaling

**Fig. 1:**
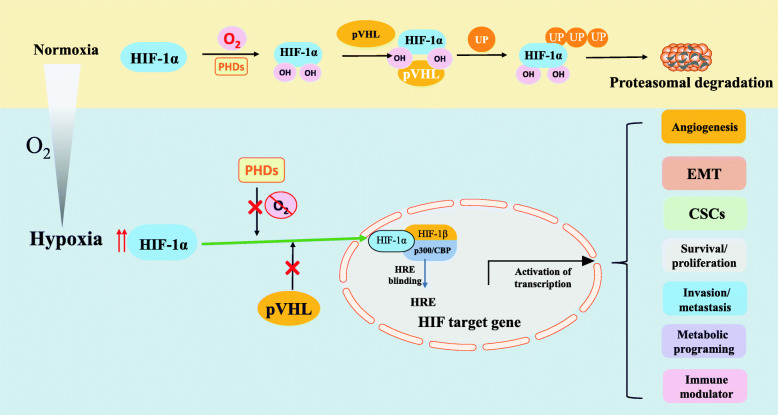
Regulation of HIFs in hypoxic TME. In oxygenated conditions, HIF is hydroxylated on proline residues by PHDs, and then binds to pVHL, catalyzing its ubiquitination-dependent proteasomal degradation. In hypoxic conditions, PHDs inhibition allows HIF-α stabilization and translocation to the nucleus, where it heterodimerizes with HIF-1β. Then the HIF-1α/1β dimer binds to the transcriptional coactivator p300/CBP and HREs, activating transcription of various HIF target genes involved in angiogenesis, EMT, CSCs, metabolic reprogramming, tumor cell survival/proliferation, invasion/metastasis, and immune regulation

The hypoxic TME is defined as a condition where partial O_2_ pressure is below 10 mmHg [13]. The establishment of hypoxic regions arises from the imbalance due to increased oxygen consumption by rapidly proliferating tumor cells, combined with the inadequate oxygen supply by abnormal tumor angiogenesis [13]. Although tumor proliferation can stimulate angiogenesis, the distribution of the tumor vasculature network is irregular, and tumor cells are typically farther from the nearest capillary than cells in normal tissues [15]. Consequently, diffusion limits, leakiness, and malformation of the tumor vasculature contribute to the hypoxic milieu. At the molecular level, the adaption of tumor cells to the hypoxic TME is largely mediated by the hypoxia-inducible factor (HIF) family of transcription factors, although HIF-independent mechanisms have also been described (Fig. 1) [16]. HIFs are heterodimeric helix-loop-helix proteins consisting of an O_2_-sensitive α-subunit (HIF-1α, HIF-2α, and HIF-3α) and a constitutively expressed β-subunit (HIF-1β) [17]. HIF-1α and HIF-2α have crucial roles in the positive hypoxic response, whereas HIF-3α is considered a negative regulator [7]. In normoxic conditions, the conserved proline residues of HIF-1α, following hydroxylation by prolyl-hydroxylases (PHDs), bind to the Von Hippel Lindau tumor suppressor protein (pVHL), catalyzing its ubiquitination-dependent proteasomal degradation [13]. However, once oxygen deprived, the inhibition of PHDs allows HIF-1α accumulation and translocation to the nucleus, where it heterodimerizes with HIF-1β. The heterodimer HIF-1α/HIF-1β then binds to the transcriptional coactivator p300/CBP and the hypoxia-responsive element (HRE) and activates HIF target gene transcription [16]. HIF-1α and HIF-2α have similar DNA binding and dimerization domains, but different transactivation domains, suggesting that they have both overlapping and distinct target genes and regulation mechanisms [18]. Hypoxia-dependent HIF-1α and HIF-2α allow for the induction of numerous target genes regulating various biological processes of tumors, including angiogenesis, the epithelial-mesenchymal transition (EMT), maintenance of cancer stem cells (CSCs), metabolic reprogramming, tumor cell survival/proliferation, invasion/metastasis, and immune regulation [7, 16].

## Hypoxia dampens the antitumor immune response

**Fig. 2:**
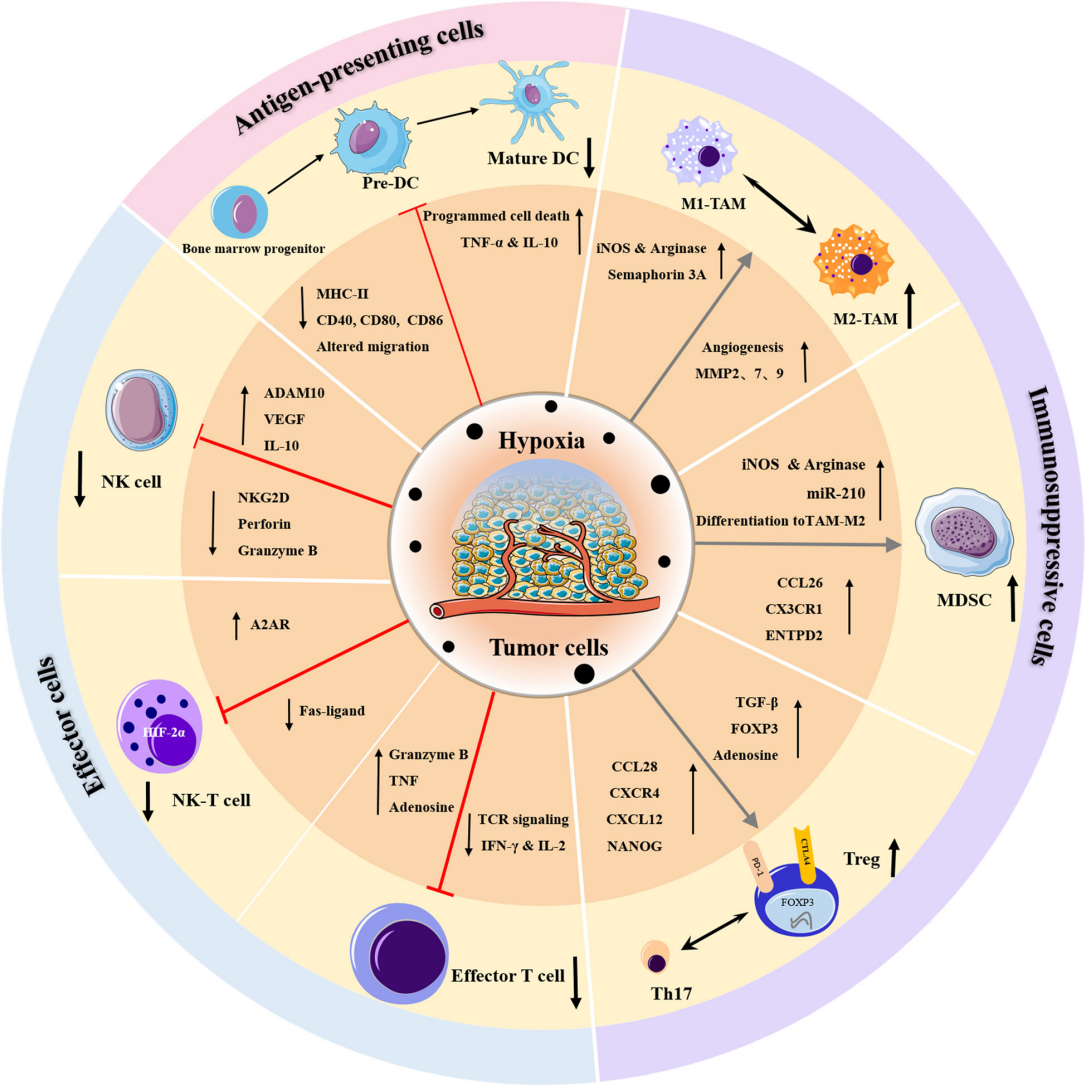
Graphical summary that explains the effects of hypoxia on key immune cellular populations in the TME. Hypoxia can influence the diverse aspects of tumor immunity. In general, Hypoxia can induce immune tolerance and immune escape by interfering with survival, migration and fuctions of effector cells, including effector T cell, NK-T cells, and NK cells, supporting immunosuppressive cells (Tregs, MDSCs, TAMs), and causing an increase in the production of immunosuppressive cytokines and a decrease in the release of effector cytokines

Hypoxia and HIFs have been proposed to regulate diverse aspects of tumor immunity, especially immune cell populations that are essential to exert effective antitumor immune responses. Destruction of any of these cell populations can weaken the immune response and allow tumors to escape detection and immune-mediated killing. In this section, we highlight some of the key immune cell populations and how their differentiation and function are altered under hypoxic conditions (Fig. 2).

### Effect of hypoxia on effector cells

#### Hypoxia interferes with the cytotoxic activity of effector T lymphocytes

Effector T lymphocytes are dominant cellular components of the adaptive immune response to tumor neoantigens. In vitro experiments have indicated that low concentrations of oxygen significantly depressed T lymphocyte proliferation and function compared with normal oxygen conditions [19]. Since there are large hypoxic regions in the spleen and lymph nodes, this probably has a physiological role to prevent CD8+ T-cell activation by stabilizing HIF1-α and suppressing TCR-mediated Ca^2+^ signaling [20]. Hypoxia has been reported to promote apoptosis of T lymphocytes, and very few T cells are found around hypoxia-induced necrotic regions of solid tumors [21]. Hypoxia can delay the differentiation of effector cells and decrease the production of effector and proliferative cytokines such as interferon-γ (IFN-γ) and interleukin-2 (IL-2). However, hypoxia has also been shown to augment the lytic capacities of CD8+ T cells by the secretion of effector molecules such as granzyme B and tumor necrosis factor (TNF) in mouse models [22]. Deletion of the HIF-1α gene in activated T cells has been shown to enhance T-cell responses by increasing proliferation and IFN-γ production, suggesting that HIF-1α is an immunosuppressive regulator of T-cell function under hypoxic conditions [23]. On the contrary, other studies indicated that hypoxia prevents cell death induced by T-cell activation and improves tumor survival. A recent study suggested that HIF-1α-deficient T cells have normal proliferation and Th-1/Th-2 polarization [24], whereas HIF-1α deficiency in murine thymocytes was shown to lead to increased caspase-8-mediated apoptosis [25]. In addition, hypoxia-driven metabolic alterations may affect the function of T cells and even their survival. In hypoxic conditions, increased glycolysis and the action of proton transporters and carbonic anhydrases increase the accumulation of lactic acid and adenosine in the TME [26]. High levels of lactate have been proven to block the mechanistic target of rapamycin (mTOR) pathway, suppress glycolysis, and impair T-cell proliferation and effector functions [17]. Interaction of free adenosine with the adenosine A2A receptor (A2AR) on the T cell surface leads to the accumulation of cyclic adenosine monophosphate (cAMP) and inhibition of T cell proliferation and cytotoxicity [26]. Overall, hypoxia can be considered to be a negative regulator of the T-cell effector response, which can contribute to immunosuppression in the TME.

#### Hypoxia reduces functions of natural killer and natural killer T-cells

Natural killer (NK) cells are a class of innate cytotoxic lymphocytes that can kill target cells without prior sensitization. It has been shown in multiple models that hypoxia can mediate suppression of NK cytotoxic effects [11]. The direct impact of hypoxia on NK cells may involve HIF-1α upregulation via the activation of the phosphatidylinositol 3 kinase (PI3K)/mTOR signaling pathway [27]. Furthermore, hypoxic tumor cells, via HIF-1α, can upregulate the expression of the metalloproteinase ADAM10, which is responsible for the shedding of the natural killer group 2, member D (NKG2D) ligand major histocompatibility complex (MHC) class I chain-related molecule A (MICA) from the tumor cell surface [28]. Soluble MICA can downregulate the expression of the NKG2D activating receptor on NK and T cells, resulting in tumor evasion from the immune system [28]. Moreover, hypoxia can downregulate the expression and function of the major activating NK-cell receptors (NK p30, NK p44, NK p46, and NKG2D). This can result in impaired tumor cell killing, without affecting the Fc-γ receptor CD16, which can trigger antibody-dependent cellular cytotoxicity [29]. The decreased expression of the activating NKG2D receptors and the induction of granzyme B and intracellular perforin by hypoxia has also been described in hematopoietic tumors [11]. Furthermore, hypoxia can induce regulatory T cell (Treg) infiltration, which can activate the immunosuppressive cytokine transforming growth factor β (TGF-β) to hinder NK cell functions [30]. Krzywinska et al. [31] demonstrated that the hypoxic response in NK cells could inhibit vascular endothelial growth factor (VEGF)-driven angiogenesis and enhance functionally improved vessels to promote tumor growth. The regulatory effects of hypoxia on NK-T cells has been less well investigated; however, HIF-2α appears to be involved in multiple immune suppression pathways in NK-T cells [32]. Zhang et al. [32] reported that increased HIF-2α could suppress NK-T cell activation by downregulating the expression of Fas-ligands and simultaneously inducing A2AR expression.

### Effects of hypoxia on dendritic cells

As dominant antigen-presenting cells, dendritic cells (DCs) capture tumor antigens and present them to antigen-specific T cells via MHC glycoproteins, thus activating naive T cells and initiating specific immune responses. The effects of the hypoxic TME on the differentiation and function of DCs have been widely studied. Hypoxia has been shown to reduce circulating plasmacytoid DCs with a corresponding increase in the expression of cytokines such as TNF-α and IL-6 [33]. Immature DCs exposed to the hypoxic TME express high levels of HIF-1α along with the upregulation of B-cell lymphoma 2 (Bcl-2)/Adenovirus E1B 19-kd interacting protein 3 (BNIP3), which mediate programmed cell death in these DCs [34]. Numerous studies have reported that hypoxia decreases the surface expression of DC differentiation and maturation markers, including MHC-II and co-stimulatory molecules (CD40, CD80, and CD86) [35]. Similarly, because of the downregulation of MHC-II, co-stimulatory molecules, and Th1 cytokines, hypoxia-differentiated DCs adopt a Th2-stimulating phenotype and exhibit impaired T-cell-stimulatory activities [30]. Moreover, hypoxia could impair mature DC migration to the lymph nodes via the downregulation of CCR7 by HIF-1α-dependent mechanisms [36]. Hypoxia has also been shown to decrease monocyte-derived DC chemotaxis to CCR4 and CCR5 ligands [30]. In line with this, hypoxic DCs have been shown to alter the chemokine receptor (CCR2, CCR3, and CXCR4) expression profile and promote the upregulation of proinflammatory cytokines such as IL-1β and TNFα [30]. Furthermore, hypoxic DCs secrete large amounts of osteopontin, whose function is to enhance the migration of tumor cells [37]. Several studies have demonstrated that the upregulation of cytokines such as IL-10 and VEGF under hypoxic conditions can inhibit the differentiation and maturation of DCs. It has been reported that IL-10 prevents monocyte differentiation into DCs while promoting their differentiation into mature macrophages [38]. A study by Takayama et al. [39] revealed that IL-10 enhances CCR5 but downregulates CCR7 expression by DCs, thereby impairing chemotactic responses and homing ability. Data from a mouse model indicated that the injection of recombinant VEGF into tumor-free mice results in suppressed development of DCs related to Gr-1+ immature myeloid-derived suppressor cell (MDSC) accumulation, leading to an inhibition of T cell functions [25]. On the contrary, other studies have shown that hypoxia can induce differentiation and maturation of DCs. Hypoxia in combination with lipopolysaccharide markedly increased the expression of costimulatory molecules, promoted the synthesis of proinflammatory cytokines, and induced the proliferation of allogeneic lymphocytes. This DC activation was accompanied by accumulation of HIF-1α protein levels, induction of glycolytic HIF target genes, and enhanced glycolytic activity [40]. In mature DCs, hypoxia increase the expression of genes involved in the innate and adaptive immune responses, thus promoting T cell activation [30, 41]. Generally, hypoxia is thought to influence the differentiation and function of DCs, thereby inhibiting the activation of T cells.

### Hypoxia induces immunosuppressive cells contributing to immune tolerance

#### Hypoxia drives immunosuppressive function of regulatory T cells

Tregs are a specialized subset of CD4 T cells, which are identified by the expression of the FOXP3 gene, and are responsible for immune suppression and tumor tolerance by the production of TGF-β and suppression of effector T cells. Hypoxia-driven HIF-1 can directly bind to the FOXP3 promoter region in CD4+ T cells and promote the transcription of *Foxp3* in a TGF-β-dependent mechanism and enhance the differentiation to Tregs [42]. Interestingly, conditional VHL deletion in regulatory Foxp3+ T cells can lead to constitutive stabilization of HIF-1α and conversion to a Th1-like phenotype, with an overproduction of Treg IFN-γ, and impairment of Treg suppressive activities [43]. Another study suggested that HIF-1α could promote proteasomal degradation of FOXP3, while HIF-1α combined with IL-6 promotes the formation of Th17 cells [24]. These findings indicated that different microenvironments with diverse cytokines lead to differential effects of HIF-1α on FOXP3 and Treg function. Hypoxic tumors also attract Tregs into the TME via impacting the cytokine profile. Indeed, a previous study on ovarian cancer suggested that hypoxia increases the secretion of CCL28 to enhance the recruitment of Tregs, which in turn promotes angiogenesis and tumor tolerance [44]. Similarly, in hepatocellular carcinoma, hypoxia was shown to promote the recruitment of Tregs by upregulating CCL28 in a HIF-1α-dependent manner [45]. By binding to its receptor, CCR10, CCL28 can effectively recruit CCR10+ Treg cells to the tumor site, thus suppressing the functions of effector T-cells and promoting the tumor growth [44, 46]. Furthermore, Treg recruitment in basal-like breast tumors is associated with hypoxia-induced CXCR4 upregulation in Tregs, which is also related to CXCL12 expression [47]. We have also provided evidence that hypoxia-induced NANOG, a transcription factor associated with stem cell self-renewal, in tumor cells can directly bind to the TGF-β1 promoter to activate TGF-β1 expression. Moreover, inhibition of NANOG in melanoma cells downregulates the secretion of TGF-β1, resulting in decreased macrophage and Treg recruitment and increased CD8+ T cells [48].

#### Hypoxia influences the differentiation and function of myeloid-derived suppressor cells

Myeloid-derived suppressor cells (MDSCs) are a heterogenous population of immature myeloid cells, which can be classified into two main subtypes: polymorphonuclear (PMN-MDSC) and monocytic (M-MDSC) [49]. Besides directly repressing DCs, NK cells, and T cells and promoting immune tolerance, MDSCs also contribute to angiogenesis and metastases [50]. HIF-1α has been demonstrated to be responsible for the differentiation and function of MDSCs in the hypoxic TME. Tumor MDSCs can restrain the activities of both non-specific and antigen-specific T-cells, which are more immunosuppressive than MDSCs, in the spleen that only suppresses antigen-specific CD8+ T cells; this is mostly due to the increased arginase activity and nitric oxide (NO) production as a result of regulation by HIF-1α [49]. Hypoxia is an important driver of MDSC accumulation in the TME. In the oxygen-deprived TME, hypoxia-driven HIF can induce the recruitment of CX3CR1-expressing MDSCs by activating the transcription of *CCL26* in tumor cells. Pharmacological blockade of HIF using digoxin or antibody inhibition of CX3CR1 have been demonstrated to suppress MDSC recruitment, angiogenesis, and tumor growth [51]. Furthermore, the hypoxic TME induces overexpression of ectonucleoside triphosphate diphosphohydrolase 2 (ENTPD2), which converts extracellular ATP to 5′-AMP through stabilizing HIF-1, preventing differentiation of MDSCs and therefore promoting maintenance of MDSCs [52]. Under hypoxic conditions, HIF-1α directly binds to the HRE located in the promoter of microRNA (miR)-210; this causes miR-210 overexpression and increased MDSC-mediated T-cell repression though increased arginase activity and NO production [53]. Additionally, hypoxia promotes the differentiation of MDSCs into immune suppressive tumor-associated macrophages (TAMs) in a HIF-1α-dependent manner, further supporting the establishment of an immunosuppressive network [49]. Another study demonstrated that hypoxia decreases signal transducer and activator of transcription 3 (STAT3) activity in MDSCs via the activation of CD45 tyrosine phosphatase independently of HIF-1α, which promotes the differentiation of M-MDSCs into TAMs [54].

#### Hypoxia attracts and retains tumor-associated macrophages

TAMs constitute the primary immune cell infiltrate in the TME of solid tumors [55]. TAMs can be classified into the canonical M1-like phenotype (antitumor and proinflammatory) and the alternative M2-like phenotype (protumor and anti-inflammatory) [55]. A high level of total TAMs, especially M2 TAMs, is believed to be closely related to worse clinical prognosis in multiple malignant tumors by promoting immunosuppression, angiogenesis, tumor cell activation, and metastasis [56]. Transcriptomic analyses have demonstrated that TAMs co-express a mixture of both tumor-type specific M1 and M2 markers [57]. TAM polarization is greatly affected by the biologically complex TME. According to their position in tumors, TAMs can either adopt an M2-like proangiogenic and immunosuppressive phenotype in hypoxic niches or an M1-like proinflammatory phenotype in normoxic regions [56]. This spatial distribution is thought to be one such way that hypoxia and HIF-1α induce the expression of migratory stimulating factors, including VEGF, EMAP-II, SDF1α, endothelin, and eotaxin, and facilitate the recruitment and entrapment of immature myeloid cells into the TME. Various factors enriched in this environment, including prostaglandin E2, TGF-β, VEGF, IL-4, IL-6, and ROS, are favorable for the differentiation of macrophages into suppressive M2 TAMs [11]. Additionally, hypoxic tumor cells produce lactic acid mainly by anaerobic glycolysis, resulting in induction of M2-associated genes to promote the differentiation of macrophages into M2 TAMs by several mechanisms [52]. high concentration of lactate within the anaerobi TME increases VEGF expression and the M2-like polarization of TAMs, which is partly mediated by HIF-1α activation [58]. lactate can also decrease lysosomal degradation of HIF2α by actively mTOR-mediated downregulation of Atp6v0d2 expression in TAMs, thus inducing expression of its target genes including VEGF and M2-like homeostatic genes like *Mrc1, Retnla* and *Arg1* [59]. Strikingly, lactate-derived histone lysine lactylation as a new epigenetic modification can directly stimulates gene transcription from chromatin [60]. Emerging evidence shows that progressive accumulation of lactate and histone lactylation at gene promoters with delayed kinetics serves as a ‘lactate clock’, which promotes a late-phase switch to a homeostatic phenotype and the expression of genes with M2-like functions after stimulation [61]. Recently, Semaphorin 3A (Sema3A), upregulated by HIF-1α, was reported to mediate the migration of TAMs into the hypoxic TME via Neuropilin-1 binding [62]. After the migration, HIF-1α was shown to downregulate Sema3A, retaining TAMs in the hypoxic areas [11]. Blockade of the Sema3A/Neuropilin-1 pathway could repress tumor growth and metastasis by preventing angiogenesis and restoring antitumor immunity [62]. Additionally, HIF-1α-dependent hypoxia can increase macrophage-mediated T cell suppression. In a hypoxic breast cancer model, myeloid HIF-1α was shown to mediate the induction of T cell suppression by the control of arginase-I (ArgI) and inducible NO synthase (iNOS) in macrophages [63]. Hypoxic macrophages directly upregulate angiogenic molecules such as angiopoietin, VEGF, VEGFR, FGF2, IL-8, and CXCL8 to promote angiogenesis. Proteolytic enzymes released by macrophages destroy the extracellular matrix and lead to tumor invasion. In the hypoxic TME, tumor cells promote the expression of endothelin 1 and 2 while concomitantly promoting matrix metalloproteinase (MMP)2 and MMP9 release from macrophages [34]. TAMs can also secrete MMP7 in hypoxic regions of tumors, which can cleave Fas-ligand from the neighboring cells, and form a soluble decoy, protecting tumor cells from Fas-ligand-mediated killing by T and NK cells [34].

### Hypoxia-driven alteration of immune checkpoints

**Fig. 3:**
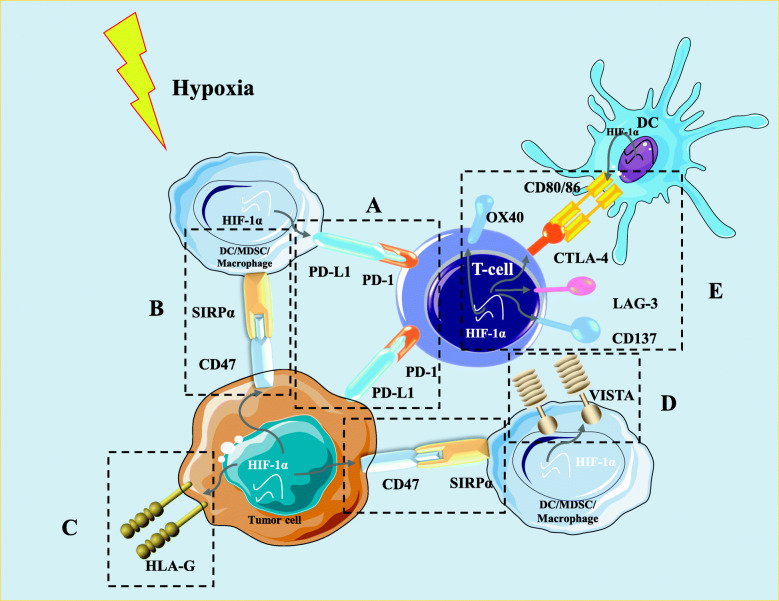
The regulation of hypoxia on immune checkpoints in the TME. **a** Under hypoxia, the stabilization of HIF-1α upregulates the expression of PD-L1 in hypoxic tumor cells DCs, macrophages, and MDSCs. **b** HIF-1α is involved in the upregulation of CD47 on tumor cell surface. The binding of CD47 to SIRPα, abundantly expressed on myeloid-linage hematopoietic cells like MDSCs and TAMs, delivers a potent “don’t eat me signal” to block phagocytosis of cancer cells. **c** Hypoxia upregulates the expression of HLA-G on the surface of tumor cells. The upregulated HLA-G binds to its inhibitory receptors on immune cells, inducing immune suppression and promoting immune escape by interfering with DC antigen presentation, suppressor T-cell induction, and cytotoxic attack inhibition. **d** Hypoxia can upregulate the expression of VISTA on myeloid cells, including MDSCs, macrophages, and DCs by the binding of HIF-1α to the HRE in VISTA promoter, suppressing T cell proliferation and activity. **e** Through HIF-1α stabilization, hypoxia upregulates the expression of inhibitory immune checkpoints (LAG-3 and CTLA4) and the co-stimulatory factors (CD137and OX40) on the surface of T cells, and decreased co-stimulatory molecules like CD40, CD80, and CD86 expressed on the surface of DCs

In addition to the regulatory mechanisms mentioned above, recent evidence supported the ability of hypoxia to regulate immune checkpoints, which may contribute to the establishment of the immunosuppressive TME. Several important immune checkpoint molecules, including PD-L1, human leukocyte antigen G (HLA-G), cluster of differentiation 47 (CD47), and V-domain Ig suppressor of T-cell activation (VISTA), are regulated by hypoxia and contribute to immune evasion (Fig. 3).

One key mechanism by which tumor cells attenuate antitumor immunity is via the expression of PD-L1 and its interaction with its inhibitory receptor PD-1, expressed on activated T lymphocytes and other immune cells. Indeed, studies have indicated that hypoxia can upregulate the expression of PD-L1 in tumor cells, DCs, tumor-infiltrating macrophages, and MDSCs [30]. Moreover, it has been shown that hypoxia via HIF-1α can increase the levels of PD-L1 protein in human breast and prostate carcinoma cells [64]. Data from B16-F10 melanoma-bearing mouse models have revealed that the exposure of MDSCs isolated from the spleen to hypoxia significantly promotes PD-L1 expression [65]. Further study showed that HIF-1α binds to the HRE in the proximal promoter of the PD-L1 gene upon its stabilization in hypoxic cells [65]. Under hypoxic conditions, inhibition of PD-L1 promotes MDSC-mediated T cell activation along with decreased MDSC production of IL-6 and IL-10 [65]. HIF-2α is also involved in PD-L1 upregulation, and in patients with clear cell renal cell carcinoma, increased PD-L1 expression is strikingly correlated with VHL mutation and HIF-2α stabilization [66]. According to the analysis of samples from paragangliomas and pheochromocytomas, PD-L2 expression but not PD-L1 expression is significantly correlated with strong hypoxia-driven HIF-1α and carbonic anhydrase 9 (CAIX) [67]. These data support that simultaneous inhibition of HIF-1α and PD-L1/PD-L2 may be a potent approach to improve cytotoxic T cell activity.

HLA-G, a non-classical MHC-I molecule, is considered another immune checkpoint marker with relevance to immunotherapy [68]. The abnormal expression of HLA-G on malignant tumors has been found to correlate with a high invasive or metastatic status and poor clinical outcome [69]. The binding of soluble or membrane-bound HLA-G to its inhibitory receptors on immune cells can induce immune suppression and promote immune escape by interfering with DC antigen presentation, suppressor T-cell induction, and inhibition of cytotoxic effects [68]. Upregulation of HLA-G by hypoxia at the mRNA level has been confirmed previously [70]. A study by Mouillot et al. firstly demonstrated that HIF-1α upregulates the expression of HLA-G mRNA in the HLA-G–negative M8 melanoma cell line under hypoxic conditions [70]. Nevertheless, hypoxia decreases the transcriptional activity and protein level of HLA-G in constitutively expressing HLA-G cell lines. This may be due to the fact that hypoxia maintenance might result in the channeling of cell energy into the expression of productive genes at the cost of HLA-G [70]. A few HREs have been identified in the HLA-G promoter, which suggests that the mechanism underlying hypoxia-dependent expression of HLA-G most likely depends on the binding of HIF-1 to HRE motifs and the induction of HLA-G transcription [69]. Yaghi et al. [71] found that the expression of the HLA-G gene in glioma cells is mediated by HIF-1α binding to the HRE motif in exon 2; however, the effects of hypoxia on other MHC-I molecules in tumors are less well studied. Additionally, studies have confirmed that HLA-E in human tumor cells and Qa-1 in mouse tumor cells could be significantly upregulated when simultaneously exposed to oxygen and glucose deprivation. This leads the cells to interact with the inhibitory CD94/NKG2 receptor on activated T cells and allows escape from CD8+ T-cell recognition [72].

CD47, also called integrin-associated protein, is a transmembrane immune checkpoint protein on the surface of solid and hematologic cancer cells, whose overexpression is related to poor clinical prognoses [73]. The major mechanism for CD47-mediated immune escape is through the interaction of CD47 with signal regulatory protein α (SIRPα), which is abundantly expressed on myeloid-lineage hematopoietic cells such as MDSCs and TAMs. This interaction causes SIRPα phosphorylation to deliver a potent “don’t eat me signal,” blocking phagocytosis of cancer cells [73]. Recent investigations have noted that HIF-1α can directly bind to its promoter to regulate the transcription of CD47 gene, and inhibition of CD47 increases the phagocytic ability of macrophages against breast tumor cells [74]. In pancreatic adenocarcinoma, hypoxia-upregulated CD47 expression blocks prophagocytic signals in both macrophages and MDSCs [75]. Additionally, data from various pre-clinical models indicated that CD47 blocking antibodies significantly increases the expression of antigen-specific CD8+ T cells and promotes T cell-mediated tumor cell killing [76]. Thus, the CD47-SIRPα axis is not only a negative checkpoint for innate immunity but also impairs adaptive immunity. Indeed, the CD47-SIRPα axis has become a promising target in cancer immunotherapy, and anti-CD47 antibody is currently investigated for use in various solid tumors [73].

A recent study found that VISTA, a negative checkpoint regulator of the B7 family, is overexpressed in the hypoxic TME of colorectal cancer mouse models and patients [13]. VISTA is preferentially expressed on myeloid cells, including MDSCs, macrophages, and DCs, in the hypoxic TME as a consequence of the binding of HIF-1α to the HRE in the VISTA promoter [49]. Hypoxia-induced VISTA expression may suppress T cell proliferation and activity [13]. Additionally, HIF-1α has been shown to be related to the overexpression of co-stimulatory and co-inhibitory receptors on hypoxic lymphocytes, including CD137, OX40, CTLA-4, and LAG-3, compared with lymphocytes under normoxic conditions [77].

### Hypoxia-impaired susceptibility of tumor cells to effector-mediated cytotoxicity

**Fig. 4:**
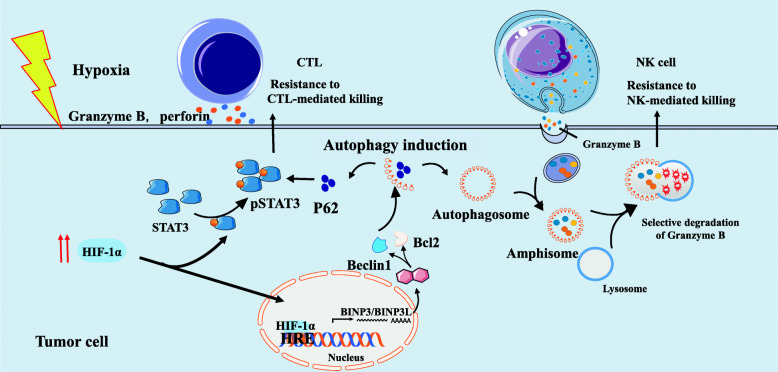
Hypoxia-impaired susceptibility of tumor cells to effector-mediated killing. Under hypoxia conditions, tumor cells stabilize HIF-1α and activate pSTAT3, which renders them resistant to CTL-mediated lysis. Hypoxia-induced autophagy is responsible for the acquisition of resistance to CTL-mediated killing by inhibiting the degradation of pSTAT3. Similarly, in hypoxic tumor cells, excessive autophagy causes fusion of autophagosome with gigantosome to form amphisome. Subsequently, fusion of amphisome with lysosome triggers selective degradation of granzyme B, making hypoxic tumor cells resistant to NK cell-mediated killing

It has been increasingly appreciated that hypoxia-induced autophagy alters the susceptibility of tumors to immune cell attack (Fig. 4). Autophagy is a cellular catabolic process that functions to maintain intracellular metabolic homeostasis by the capture and degradation of impaired organelles and misfolded proteins in response to stress conditions like hypoxia [78]. The negative impact of hypoxia-induced autophagy in tumor cells is mediated by the nuclear translocation of HIF-1α. Hypoxia-dependent induction of HIF-1α can upregulate expression of BNIP3/BNIP3-like (BNIP3L), which in turn can activate autophagy by preventing the combination of Beclin1 with Bcl-2 [78]. Recently, hypoxia-induced autophagy has been reported to impair CTL-mediated tumor cell lysis by the regulation of phospho-STAT3 (pSTAT3) in target cells. Inhibition of autophagy correlates with decreased hypoxia-induced phosphorylation of STAT3 and renders lung cancer cells sensitive to T cell cytotoxicity via a mechanism involving the ubiquitin proteasome system and SQSTM1/p62 [79]. Furthermore, Noman’s group found that targeting autophagy with hydroxychloroquine in hypoxic tumors could improve the efficacy of TRP-2-peptide vaccination and promote tumor regression in vivo [80]. Moreover, other studies have shown that upregulation of NANOG mediates hypoxia-induced resistance to cytotoxic lymphocyte (CTL) lysis, while knockdown of NANOG in hypoxic tumor cells significantly restores CTL-dependent tumor cell killing [81]. Interestingly, NANOG depletion leads to inhibition of STAT3 phosphorylation and nuclear translocation [81]. NANOG binds directly to the enhancer sequence of BNIP3L, activates its transcription, and contributes to autophagy under hypoxia [82]. HIF-1α also mediates the susceptibility of tumor cells to CTL-mediated lysis by the upregulation of miR-210 [13]. Indeed, coordinate silencing of miR-210 target genes, including tumor protein p53-inducible protein 11 (*TP53I11*), homeobox A1 (*HOXA1*), and non-receptor protein tyrosine phosphatase type 1 (*PTPN1*), dramatically decreases tumor cell susceptibility to CTL-mediated lysis [13].

The role of hypoxia-induced autophagy has been extended to the regulation of NK cell-mediated innate antitumor immunity. There is evidence that hypoxic tumor cells can evade NK-mediated immune surveillance via activating autophagy [83]. Hypoxic tumor cells can use autophagy to their advantage and selectively degrade the NK cell-released granzyme B, which is responsible for NK-mediated apoptosis of target cells [83]. A study exploring the role of autophagy in the regulation of NK cell-mediated immune response demonstrated that the VHL-mutated 786-O renal carcinoma cell line is resistant to NK-mediated lysis [84]. It was found that 786-O cells express more inositol 1,4,5-triphosphate receptor type 1 (ITPR1) than a VHL-corrected cell line (WT7), which could regulate NK-mediated killing by activating autophagy in target cells [84]. Furthermore, inhibition of ITPR1/autophagy in tumors significantly increases granzyme B activity and improves NK-mediated tumor extinction [84, 85].

Taken together, these data provide that regulation of hypoxia on immune cells ought to play a largely negative role in modulating tumor immunity. Hypoxia seems to interferes with immune cell populations exerting effective antitumor immune responses while facilitating immunosuppressive cells in terms of their differentiation, recruitment, and suppressive functions, as well as remodeling the metabolic landscape to support immune tolerance. Importantly, hypoxia stress modulates the expression levels for important molecular targets in cancer immunotherapy including PD-L1, HLA-G, CD47, and VISTA. In-depth research of this intricate balance will contribute to improve the efficacy of cancer immunotherapy.

## Targeting hypoxia to enhance the efficacy of immunotherapy

Currently, available immunotherapies in the clinic have not yet considered the impact of the hypoxic TME. A variety of strategies have been proposed to overcome hypoxia in cancer; among them, the majority of studies have involved hypoxia-activated prodrugs (HAPs), inhibition of HIF signaling, downstream targeting of important hypoxic pathways, like the UPR (unfolded protein response) and mTOR pathways, and metabolic intervention [86]. Others, including siRNA-mediated gene therapies and recombinant anaerobic bacteria, are at relatively early phases of development. Obviously, due to its central role in regulating tumor progression and immune suppression, it is conceivable that hypoxia may be considered as a potential target in combined cancer immunotherapy. In this section, we discuss the potential of manipulating hypoxia to improve the efficacy of cancer immunotherapy based on the data obtained from both pre-clinical and clinical studies (Table 1).

**Table 1:** Summary of Hypoxia-targeting agents with potential to improve immunotherapy

Agents	Target	Effect	Reference
Evofosfamide (TH-302)	Hypoxia activated prodrug	Upon activation in oxygen deficient regions, evofosfamide is converted selectively to the drug’s active form, dibromo isophosphoramide mustard, a potent alkylator	[87]
Praziquantel (EO9)	Hypoxia activated prodrug	Apaziquone can be converted to a cytotoxic species after enzymatic activation	[88]
SN30000	Hypoxia activated prodrug	Improved tirapazamine analogue with potential for targeting tumor hypoxia in humans	[27]
Doxorubicin	Targeting HIF DNA binding	Inhibits binding of HIF-1 to the HRE sequence	[12]
Daunorubicin	Targeting HIF DNA binding	Inhibits binding of HIF-1 to the HRE sequence	[12]
Anti-sense HIF-1a therapy	Anti-sense HIF-1a	Engineered down-regulation of HIF-1a by gene transfer of an antisense HIF-1a plasmid leads to the down-regulation of VEGF, and decreased tumor microvessel density.	[89]
PX-478	HIF-1α inhibitor	Reduces expression of Foxp3 and VEGF transcript and/or protein, molecules that are directly controlled by HIF-1	[90]
CRLX101	HIF-1α inhibitor	Suppresses HIF-1α as well as topoisomerase 1	[91]
POM-1	ENTPD2 inhibitor	Depletes MDSCs and mitigates cancer growth	[52]
anti-CAIX antibodies	Targeting CA IX	Mediates immune killing of CAIX+ tumor cells	[92]
SLC-0111	CA IX inhibitor	Decreases glycolytic metabolism of tumor cells and acidification of the TME	[93]
SCH58261	A2AR antagonist	Inhibits immunosuppressive adenosine	[94]
Oxygen therapy	Supplementing oxygen	Decreases the tumor hypoxia and HIF-1α-CD39/CD73-driven extracellular adenosine accumulation	[95]
Metformin	Inhibiting oxygen consumption	Inhibits both oxygen consumption and subsequent tumor hypoxia	[96]
Bevacizumab	Inhibiting the binding of VEGF to its receptors	Anti-angiogenesis	[97]
Lenvatinib	Multi-kinase VEGFR inhibitor	Anti-angiogenesis	[98]
Cabozantinib	Tyrosine-kinase inhibitor	Anti-angiogenesis	[38]

### Hypoxia-activated prodrugs

HAPs, also known as bioreductive prodrugs, are a category of biologically inactive compounds, which can be transformed into pharmacologically active substances through enzymatic reduction to selectively kill tumor cells in the hypoxic TME [99]. Despite displaying high hypoxic cytotoxicity according to pre-clinical data, several HAPs have shown disappointing clinical efficacy, and their development has been discontinued as a result [99]. However, evofosfamide (TH-302) has been shown to be non-lymphotoxic and can be applied concurrently with immunotherapy without damaging the T-cell-mediated antitumor response in animal and clinical studies [13]. It has been reported that TH-302 therapy in combination with blockade of PD-1 and CTLA-4 can cure more than 80% of tumors and significantly extend survival in prostate-derived mouse models. It is believed that the success of TH-302 therapy is due to its ability to drive T cells into hypoxic tumor regions, decrease the density of MDSCs, and impair the ability of tumors to produce suppressive myeloid stroma [87]. Indeed, CD8+ T cells in the combination group showed increased granzyme B production, expression and proliferation of CD44, and production of effector cytokines [87]. In line with this, an ongoing clinical trial (NCT03098160) is being conducted to evaluate the therapeutic efficacy of TH-302 in combination with ipilimumab against several cancers, including melanoma, pancreatic, and prostate tumors. Simultaneously, the data from a recently published Phase II clinical trial on TH-302 in combination with adriamycin for the treatment of soft tissue sarcoma (or gemcitabine for pancreatic cancer) are encouraging [100]. Moreover, topical administration of praziquantel (EO9), a mitomycin C derivative prodrug, has shown efficacy in patients with superficial bladder cancer [88]. On this basis, two Phase III clinical trials (NCT00461591 and NCT00598806) have been conducted to evaluate the treatment effect of EO9 as an adjuvant therapy in patients with bladder cancer who underwent surgery. SN30000, which is a tirapazamine analogue with improved pharmacokinetic and pharmacodynamic properties, has also been shown to have antitumor effects in xenograft models [27].

### Drugs targeting HIF signaling pathways

Currently, a growing number of drugs targeting HIFs are being developed, which can be classified into modulators interfering with HIF dimerization, mRNA or protein expression, degradation, DNA binding, and transcriptional activity, according to their mode of action [101]. Since some excellent reviews have provided a comprehensive overview of these agents, we did not discuss this further in the current review [25, 101]. As discussed previously, HIFs are essential for the adaptation of tumor cells to the hypoxic TME, which provides favorable rationale for the combination of HIF pathway inhibition and immunotherapy. Many classical chemotherapeutic agents, including epirubicin, cisplatin, doxorubicin, and cyclophosphamide, are known immunogenic cell-death inducers, which, in combination with immunotherapy, may boost antitumor immune responses [102]. Previous studies have reported that downregulation of HIF-1 expression by antisense HIF1-α with B7–1-T could enhance NK cell and CD8 T cell-mediated antitumor immunity and induce rejection of tumors [89]. Moreover, HIF1-α inhibition synergized with DC-based immunotherapy was shown to result in tumor regression and improved survival by augmenting the proliferation and function of cytotoxic T lymphocytes and increasing IFN-γ production in a breast cancer model [90]. CRLX101, a dual inhibitor of topoisomerase 1 and HIF-1α, also demonstrated synergy with immunotherapy in pre-clinical models [91]. Compared with ICI monotherapy, the combination of HIF-1-mediated ectoenzyme ENTPD2 inhibitors and ICI (anti-CTLA-4/PD-1) significantly enhanced T cell infiltration into the tumor and extends the survival of tumor-bearing mice [52]. It is noteworthy that hypoxia-driven HIF pathways represent a complex network of multiple overlapping cascades, and the use of combination therapy warrants further exploration.

### Metabolic regulation

CAIX, a cell-surface pH regulatory enzyme, can be upregulated by HIF-1α and HIF-2α to activate glycolysis, and the overexpression is related to reduced immune activity in patients with various solid malignancies [38]. In the study by Marasco’s group, a monoclonal antibody specifically targeting CAIX was found to inhibit CAIX-positive tumor growth through promoting immune-mediated killing, including macrophage-mediated antibody-dependent cell-mediated cytotoxicity, NK cell-mediated antibody-dependent cell-mediated cytotoxicity, and complement-dependent cytotoxicity [92]. Furthermore, Chafe et al. [93] revealed that CAIX inhibition by a small-molecule inhibitor, SLC-0111, decreases the glycolytic metabolism of tumor cells and acidification of the TME, thereby increasing immune activity. Inhibition of CAIX makes the tumor sensitive to ICIs, leading to enhanced Th1 responses and decreased tumor growth and metastasis. These results indicate that targeting CAIX combined with immunotherapy is a promising therapeutic strategy for improving clinical outcomes in patients with hypoxic tumors.

It is known that hypoxia can induce the accumulation of extracellular adenosine via the HIF-1α-CD39/CD73-adenosine signaling pathway [103]. Indeed, high levels of extracellular adenosine can considerably alter immune cell functions and drive an immunosuppressive TME [103]. Pre-clinical study on HNSCC mouse models have shown that SCH58261,a A2AR antagonist, significantly decreases the population of CD4+ FOXP3+ Tregs and promotes a CD8+ T cell-mediated antitumor response, which lead to a delay in tumor growth [94]. This indicates that inhibition of A2AR may be an effective approach to enhance immunotherapy.

### Supplemental oxygenation

The use of supplemental oxygen as a simple means to enhance immunotherapy is appealing. It has been confirmed that oxygen therapy as an immunological co-adjuvant combined with other existing immunotherapies can decrease the tumor hypoxia and HIF-1α-CD39/CD73-driven extracellular adenosine accumulation. Together, this strategy functions to weaken the A2AR/A2BR-mediated pleiotropic cascade of immunosuppression in the hypoxic TME [12, 104]. Hatfield reported that respiratory hyperoxia with 60% oxygen enhances intra-tumoral infiltration and decreases inhibition of CTLs, promoting pulmonary tumor extinction induced by dual inhibition of PD-1 and CTLA-4 [95]. Adoptive immunotherapy combined with respiratory hyperoxia has been demonstrated to lead to the complete extinction of established MCA205 fibrosarcoma pulmonary tumors in a mouse model [95]. These findings justify the investigation of supplemental oxygen as an immunological co-adjuvant in combination with existing cancer immunotherapies and also support the development of A2AR antagonists and CD39/CD73 inhibitors as novel agents to improve immunotherapy [104]. In contrast, inhibiting both oxygen consumption and subsequent tumor hypoxia with metformin has been correlated with a better efficacy of PD-1 blockade immunotherapy [96]. Scharping et al. [96] suggested that employing metformin as a method to modulate tumor hypoxia and remodel the hypoxic TME could improve the sensitivity to anti-PD-1 immunotherapy, thus allowing improved intra-tumoral T-cell function and tumor regression.

### Vessel normalization

Hypoxia is caused by abnormal tumor vascularization and can upregulate VEGF through HIF-1, thus leading to a vicious circle [13]. Abnormal tumor vascularization can impair blood flow, aggravate hypoxia, and limit the delivery of nutrients and therapeutics, including antibodies and immune cells [18]. VEGF/VEGFR-targeted therapies not only induce anti-angiogenic effects that reduce hypoxia but also have immune-supportive roles [12]. However, monotherapy with angiogenesis inhibitors can significantly aggravate tumor hypoxia, resulting in therapeutic resistance and worse clinical outcomes [105]. Vascular normalization as a result of a low dose of anti-angiogenic agents has been proven to enhance the efficacy of immunotherapy and decrease toxicity [12]. A Phase I clinical trial involving the dual blockade of VEGF (bevacizumab) and CTLA-4 (ipilimumab) revealed increased tumor antigen recognition, tumor-associated endothelial activation, and infiltration of T-cells in melanomas [106]. Furthermore, a Phase Ib trial of a combination of lenvatinib and anti-PD-1 (pembrolizumab) in patients with unresectable hepatocellular carcinoma showed a good tolerability to combination therapy, promoting the initiation of a Phase III trial comparing the combination of lenvatinib + pembrolizumab with lenvatinib alone as a first-line treatment for advanced hepatocellular carcinoma [98]. Clinical trials investigating a combination of PD-1 blockade (nivolumab) with lenvatinib (NCT03418922) and cabozantinib (NCT03299946) are currently going on [38]. Another clinical study showed that anti-PD-L1 (atezolizumab) combined with bevacizumab increases the migration of tumor-specific T-cells and the number of intra-tumoral CD8+ T-cells [107]. Recently, the FDA has approved the combination of PD1/PD-L1 antibodies and anti-VEGF/VEGFR agents in lung and renal cancers based on benefits observed from three Phase III clinical trials (NCT02366143, NCT02684006, and NCT02853331) [97]. However, one interesting illuminated question is the impact of hypoxic stress on tumor heterogeneity. Given the temporal and spatial heterogeneity in tumor, vessel normalization and increasing oxygenation may have potential consequences on their evolution in time and response to treatments [86]. Some preclinical studies revealed tumor shrinkage after anti-VEGF therapy, others suggesting vascular normalization have been related to increased rates of tumor growth [108]. Data from clinical studies in breast cancer and glioblastoma patients treated with bevacizumab are also contradictory, with some studies reporting increased oxygenation in tumors, and others describing aggravation in intratumoral hypoxia [109]. Besides, the induction of hypoxia by anti-angiogenic agents have been proven to increase numbers of breast cancer stem cells [110]. Consequently, it is essential to induce vascular normalization according to different pro-angiogenic factors in different tumors and at different times [111]. Optimal doses of anti-angiogenic agents for vascular normalization may vary depending on patients or disease status, and reliable biomarkers will facilitate the selection of a “vascular normalizing” or “pruning” dose [111]. Over 100 clinical studies are currently investigating the combinations of immunotherapy and anti-angiogenics. Overall, approaches to combine immunotherapy with vascular normalization hold great promise for improving the therapeutic outcomes in patients with cancer.

Given that hypoxia in the TME is a driving force of tumor progression, and play a critical role in remodeling the tumor stroma and favoring the emergence of immune privilege, the prospect of combinatorial hypoxia and immunotherapy appears to be encouraging. However, we should not ignore the fact that not all patients response well to hypoxia-modifying therapy. In the era of personalized medicine, it is essential to screen for hypoxia markers such as HIF-1 in tumor to achieve more specific targeted therapies and induce a powerful anti-tumor immune response.

## Conclusions

Solid malignant tumors create an adversarial hypoxic TME, which can impair antitumor immunity and suppress the efficacy of immunotherapy. Thus, it is attractive to consider the manipulation of hypoxia in future innovative immunotherapy, and several strategies targeting hypoxia have demonstrated synergy with immunotherapy. The primary challenge ahead is to establish which of these strategies can ameliorate immune suppression caused by hypoxia without impairing antitumor immunity. More pre-clinical and clinical studies are needed to explore the optimal combination strategies as well as the most appropriate sequence and timing of these combinations to provide insight into tailored immunotherapy for patients with cancer.

## Data Availability

Not applicable.

## Abbreviations

ICIs: Immune checkpoint inhibitors
TME: Tumor microenvironment
CTLA4: Cytotoxic T lymphocyte antigen 4
PD-1: Programmed cell death protein 1
PD-L1: PD1 ligand 1
PDT: Photodynamic therapy
ROS: Reactive oxygen species
TMB: Tumor mutational burden
MSI: Microsatellite instability
dMMR: Deficient mismatch repair
HIF: Hypoxia-inducible factor
PHDs: Prolyl-hydroxylases
pVHL: Von Hippel Lindau tumor suppressor protein
HRE: Hypoxia-responsive element
EMT: Epithelial–mesenchymal transition
CSCs: Cancer stem cells
IFN-γ: Interferon-γ
IL-2: Interleukin-2
TNF: Tumor necrosis factor
mTOR: Mechanistic target of rapamycin
A2AR: Adenosine A2A receptor
cAMP: Cyclic adenosine monophosphate
NK: Natural killer
PI3K: Phosphatidylinositol 3 kinase
NKG2D: Natural killer group 2, member D
MHC: Major histocompatibility complex
MICA: Major histocompatibility complex class I chain–related molecule A
Treg: Regulatory T cell
TGF-β: Transforming growth factor β
DCs: Dendritic cells
Bcl-2: B-cell lymphoma 2
BNIP3: Adenovirus E1B 19-kd interacting protein 3
CCR: CC chemokine receptor
CXCR: C-X-C motif chemokine receptor
VEGF: Vascular endothelial growth factor
MDSC: Myeloid-derived suppressor cell
PMN-MDSC: Polymorphonuclear MDSC
M-MDSC: Monocytic MDSC
ENTPD2: Ectonucleoside triphosphate diphosphohydrolase 2
MiR: MicroRNA
NO: Nitric oxide
TAM: Tumor-associated macrophage
STAT3: Signal transducer and activator of transcription 3
Sema3A: Semaphorin 3A
ArgI: Arginase-I
iNOS: Inducible nitric oxide synthase
VEGFR: VEGF receptor
MMP: Matrix metalloproteinase
HLA-G: Human leukocyte antigen G
CD47: Cluster of differentiation 47
VISTA: V-domain Ig suppressor of T-cell activation
CAIX: Carbonic anhydrase 9
SIRPα: Signal regulatory protein α
BNIP3L: BNIP3-like
pSTAT3: Phospho-STAT3
CTL: Cytotoxic lymphocyte
TP53I11: p53-inducible protein 11
HOXA1: Homeobox A1
PTPN1: Protein tyrosine phosphatase type 1
ITPR1: Inositol 1,4,5-triphosphate receptor type 1
HAPs: Hypoxia-activated prodrugs
UPR: Unfolded protein response

## References

[1] Sanmamed MF, Chen L (2018). A paradigm shift in Cancer immunotherapy: from enhancement to normalization. Cell..

[2] Ribas A, Wolchok JD (2018). Cancer immunotherapy using checkpoint blockade. Science (New York, NY).

[3] Hegde PS, Chen DS (2020). Top 10 challenges in Cancer immunotherapy. Immunity..

[4] Jing X, Yang F, Shao C (2019). Role of hypoxia in cancer therapy by regulating the tumor microenvironment. Mol Cancer.

[5] Vaupel P, Multhoff G (2017). Accomplices of the Hypoxic Tumor Microenvironment Compromising Antitumor Immunity: Adenosine, Lactate, Acidosis, Vascular Endothelial Growth Factor, Potassium Ions, and Phosphatidylserine. Front Immunol.

[6] McDonald PC, Chafe SC, Dedhar S (2016). Overcoming hypoxia-mediated tumor progression: combinatorial approaches targeting pH regulation, Angiogenesis and Immune Dysfunction. Front Cell Dev Biol.

[7] Qiu GZ, Jin MZ, Dai JX (2017). Reprogramming of the tumor in the hypoxic niche: the emerging concept and associated therapeutic strategies. Trends Pharmacol Sci.

[8] Phung CD, Tran TH, Pham LM (2020). Current developments in nanotechnology for improved cancer treatment, focusing on tumor hypoxia. J Control Release.

[9] Zhou T-J, Xing L, Fan Y-T (2019). Light triggered oxygen-affording engines for repeated hypoxia-resistant photodynamic therapy. J Control Release.

[10] Azimi I, Petersen RM, Thompson EW (2017). Hypoxia-induced reactive oxygen species mediate N-cadherin and SERPINE1 expression, EGFR signalling and motility in MDA-MB-468 breast cancer cells. Sci Rep.

[11] Chouaib S, Noman MZ, Kosmatopoulos K (2017). Hypoxic stress: obstacles and opportunities for innovative immunotherapy of cancer. Oncogene..

[12] Abou Khouzam R, Goutham HV, Zaarour RF, et al. Integrating tumor hypoxic stress in novel and more adaptable strategies for cancer immunotherapy. Semin Cancer Biol. 2020. 10.1016/j.semcancer.2020.01.003 31927131

[13] Noman MZ, Hasmim M, Lequeux A (2019). Improving Cancer immunotherapy by targeting the hypoxic tumor microenvironment: new opportunities and challenges. Cells..

[14] Duffy MJ, Crown J (2019). Biomarkers for predicting response to immunotherapy with immune checkpoint inhibitors in Cancer patients. Clin Chem.

[15] Feng J, Byrne NM, Al Jamal W, et al. Exploiting Current Understanding of Hypoxia Mediated Tumour Progression for Nanotherapeutic Development. Cancers. 2019;11(12). . 10.3390/cancers11121989 PMC6966647 31835751

[16] Bosco MC, D'Orazi G, Del Bufalo D (2020). Targeting hypoxia in tumor: a new promising therapeutic strategy. J Exp Clin Cancer Res.

[17] Vito A, El-Sayes N, Mossman K (2020). Hypoxia-driven immune escape in the tumor microenvironment. Cells..

[18] Datta M, Coussens LM, Nishikawa H (2019). Reprogramming the tumor microenvironment to improve immunotherapy: emerging strategies and combination therapies. Am Soc Clin Oncol Educ.

[19] Vuillefroy de Silly R, Dietrich PY, Walker PR (2016). Hypoxia and antitumor CD8(+) T cells: An incompatible alliance?. Oncoimmunology.

[20] Damgaci S, Ibrahim-Hashim A, Enriquez-Navas PM (2018). Hypoxia and acidosis: immune suppressors and therapeutic targets. Immunology..

[21] Mpekris F, Voutouri C, Baish JW (2020). Combining microenvironment normalization strategies to improve cancer immunotherapy. Proc Natl Acad Sci U S A.

[22] Doedens AL, Phan AT, Stradner MH (2013). Hypoxia-inducible factors enhance the effector responses of CD8(+) T cells to persistent antigen. Nat Immunol.

[23] Reyes A, Corrales N, Gálvez NMS (2020). Contribution of hypoxia inducible factor-1 during viral infections. Virulence..

[24] Dang EV, Barbi J, Yang HY (2011). Control of T(H)17/T (reg) balance by hypoxia-inducible factor 1. Cell..

[25] Noman MZ, Hasmim M, Messai Y, et al. Hypoxia: a key player in antitumor immune response. A review in the theme: cellular responses to hypoxia. Am J Physiol Cell Physiol. 2015;309(9):569–79. 10.1152/ajpcell.00207.2015 PMC4628936 26310815

[26] Payen VL, Porporato PE, Baselet B (2016). Metabolic changes associated with tumor metastasis, part 1: tumor pH, glycolysis and the pentose phosphate pathway. Cell Mol Life Sci.

[27] Francis A, Venkatesh GH, Zaarour RF (2018). Tumor hypoxia: a key determinant of microenvironment hostility and a major checkpoint during the antitumor response. Crit Rev Immunol.

[28] Torres N, Regge MV, Secchiari F, et al. Restoration of antitumor immunity through anti-MICA antibodies elicited with a chimeric protein. J Immunother Cancer. 2020;8(1). 10.1136/jitc-2019-000233 PMC7282397 32518090

[29] Balsamo M, Manzini C, Pietra G (2013). Hypoxia downregulates the expression of activating receptors involved in NK-cell-mediated target cell killing without affecting ADCC. Eur J Immunol.

[30] Labiano S, Palazon A, Melero I (2015). Immune response regulation in the tumor microenvironment by hypoxia. Semin Oncol.

[31] Krzywinska E, Kantari-Mimoun C, Kerdiles Y (2017). Loss of HIF-1α in natural killer cells inhibits tumour growth by stimulating non-productive angiogenesis. Nat Commun.

[32] Zhang J, Han C, Dai H (2016). Hypoxia-inducible factor-2α limits natural killer T cell cytotoxicity in renal ischemia/reperfusion injury. J Am Soc Nephrol.

[33] Yilmaz A, Ratka J, Rohm I (2016). Decrease in circulating plasmacytoid dendritic cells during short-term systemic normobaric hypoxia. Eur J Clin Investig.

[34] Daniel SK, Sullivan KM, Labadie KP (2019). Hypoxia as a barrier to immunotherapy in pancreatic adenocarcinoma. Clin Transl Med.

[35] Correale P, Rotundo MS, Botta C (2012). Tumor infiltration by T lymphocytes expressing chemokine receptor 7 (CCR7) is predictive of favorable outcome in patients with advanced colorectal carcinoma. Clin Cancer Res.

[36] Köhler T, Reizis B, Johnson RS (2012). Influence of hypoxia-inducible factor 1α on dendritic cell differentiation and migration. Eur J Immunol.

[37] Blengio F, Raggi F, Pierobon D (2013). The hypoxic environment reprograms the cytokine/chemokine expression profile of human mature dendritic cells. Immunobiology..

[38] Chang WH, Lai AG (2020). The hypoxic tumour microenvironment: a safe haven for immunosuppressive cells and a therapeutic barrier to overcome. Cancer Lett.

[39] Takayama T, Morelli AE, Onai N (2001). Mammalian and viral IL-10 enhance C-C chemokine receptor 5 but down-regulate C-C chemokine receptor 7 expression by myeloid dendritic cells: impact on chemotactic responses and in vivo homing ability. J Immunol (Baltimore, Md : 1950).

[40] Brombacher EC, Everts B (2020). Shaping of dendritic cell function by the metabolic micro-environment. Front Endocrinol.

[41] Bosco MC, Pierobon D, Blengio F (2011). Hypoxia modulates the gene expression profile of immunoregulatory receptors in human mature dendritic cells: identification of TREM-1 as a novel hypoxic marker in vitro and in vivo. Blood..

[42] Clambey ET, McNamee EN, Westrich JA (2012). Hypoxia-inducible factor-1 alpha-dependent induction of FoxP3 drives regulatory T-cell abundance and function during inflammatory hypoxia of the mucosa. Proc Natl Acad Sci U S A.

[43] Lee JH, Elly C, Park Y (2015). E3 ubiquitin ligase VHL regulates hypoxia-inducible factor-1α to maintain regulatory T cell stability and suppressive capacity. Immunity..

[44] Facciabene A, Peng X, Hagemann IS (2011). Tumour hypoxia promotes tolerance and angiogenesis via CCL28 and T (reg) cells. Nature..

[45] Ren L, Yu Y, Wang L (2016). Hypoxia-induced CCL28 promotes recruitment of regulatory T cells and tumor growth in liver cancer. Oncotarget..

[46] Wu Q, Chen JX, Chen Y (2018). The chemokine receptor CCR10 promotes inflammation-driven hepatocarcinogenesis via PI3K/Akt pathway activation. Cell Death Dis.

[47] Yan M, Jene N, Byrne D (2011). Recruitment of regulatory T cells is correlated with hypoxia-induced CXCR4 expression, and is associated with poor prognosis in basal-like breast cancers. Breast Cancer Res.

[48] Hasmim M, Noman MZ, Messai Y (2013). Cutting edge: Hypoxia-induced Nanog favors the intratumoral infiltration of regulatory T cells and macrophages via direct regulation of TGF-β1. J Immunol (Baltimore, Md : 1950).

[49] Dysthe M, Parihar R (2020). Myeloid-derived suppressor cells in the tumor microenvironment. Adv Exp Med Biol.

[50] Hou A, Hou K, Huang Q (2020). Targeting myeloid-derived suppressor cell, a promising strategy to overcome resistance to immune checkpoint inhibitors. Front Immunol.

[51] Chiu DK, Xu IM, Lai RK (2016). Hypoxia induces myeloid-derived suppressor cell recruitment to hepatocellular carcinoma through chemokine (C-C motif) ligand 26. Hepatology (Baltimore, Md).

[52] Chiu DK, Tse AP, Xu IM (2017). Hypoxia inducible factor HIF-1 promotes myeloid-derived suppressor cells accumulation through ENTPD2/CD39L1 in hepatocellular carcinoma. Nat Commun.

[53] Noman MZ, Janji B, Hu S (2015). Tumor-promoting effects of myeloid-derived suppressor cells are potentiated by hypoxia-induced expression of miR-210. Cancer Res.

[54] Kumar V, Cheng P, Condamine T (2016). CD45 phosphatase inhibits STAT3 transcription factor activity in myeloid cells and promotes tumor-associated macrophage differentiation. Immunity..

[55] Vitale I, Manic G, Coussens LM (2019). Macrophages and metabolism in the tumor microenvironment. Cell Metab.

[56] Komohara Y, Fujiwara Y, Ohnishi K, et al. Tumor-associated macrophages: Potential therapeutic targets for anti-cancer therapy. Adv Drug Delivery Rev. 2016;99(Pt B):180–185. 10.1016/j.addr.2015.11.009 26621196

[57] Müller S, Kohanbash G, Liu SJ (2017). Single-cell profiling of human gliomas reveals macrophage ontogeny as a basis for regional differences in macrophage activation in the tumor microenvironment. Genome Biol.

[58] Colegio OR, Chu N-Q, Szabo AL (2014). Functional polarization of tumour-associated macrophages by tumour-derived lactic acid. Nature..

[59] Liu N, Luo J, Kuang D (2019). Lactate inhibits ATP6V0d2 expression in tumor-associated macrophages to promote HIF-2α-mediated tumor progression. J Clin Invest.

[60] Ivashkiv LB (2020). The hypoxia-lactate axis tempers inflammation. Nat Rev Immunol.

[61] Zhang D, Tang Z, Huang H (2019). Metabolic regulation of gene expression by histone lactylation. Nature..

[62] Zhu H, Wang D, Liu Y (2013). Role of the hypoxia-inducible factor-1 alpha induced autophagy in the conversion of non-stem pancreatic cancer cells into CD133+ pancreatic cancer stem-like cells. Cancer Cell Int.

[63] Zarrilli G, Businello G, Dieci MV, et al. The Tumor Microenvironment of Primitive and Metastatic Breast Cancer: Implications for Novel Therapeutic Strategies. Int J Mol Sci. 2020:21. 10.3390/ijms21218102 PMC7662409 33143050

[64] Barsoum IB, Smallwood CA, Siemens DR (2014). A mechanism of hypoxia-mediated escape from adaptive immunity in cancer cells. Cancer Res.

[65] Noman MZ, Desantis G, Janji B (2014). PD-L1 is a novel direct target of HIF-1α, and its blockade under hypoxia enhanced MDSC-mediated T cell activation. J Exp Med.

[66] Messai Y, Gad S, Noman MZ (2016). Renal cell carcinoma programmed death-ligand 1, a new direct target of hypoxia-inducible Factor-2 alpha, is regulated by von Hippel-Lindau gene mutation status. Eur Urol.

[67] Pinato DJ, Black JR, Trousil S (2017). Programmed cell death ligands expression in phaeochromocytomas and paragangliomas: relationship with the hypoxic response, immune evasion and malignant behavior. Oncoimmunology..

[68] Curigliano G, Criscitiello C, Gelao L (2013). Molecular pathways: human leukocyte antigen G (HLA-G). Clin Cancer Res.

[69] Garziera M, Scarabel L, Toffoli G. Hypoxic Modulation of HLA-G Expression through the Metabolic Sensor HIF-1 in Human Cancer Cells. J Immunol Res. 2017:4587520. 10.1155/2017/4587520 PMC5525073 28781970

[70] Mouillot G, Marcou C, Zidi I (2007). Hypoxia modulates HLA-G gene expression in tumor cells. Hum Immunol.

[71] Yaghi L, Poras I, Simoes RT (2016). Hypoxia inducible factor-1 mediates the expression of the immune checkpoint HLA-G in glioma cells through hypoxia response element located in exon 2. Oncotarget..

[72] Sasaki T, Kanaseki T, Shionoya Y (2016). Microenvironmental stresses induce HLA-E/Qa-1 surface expression and thereby reduce CD8(+) T-cell recognition of stressed cells. Eur J Immunol.

[73] Logtenberg MEW, Scheeren FA, Schumacher TN (2020). The CD47-SIRPα immune checkpoint. Immunity..

[74] Zhang H, Lu H, Xiang L (2015). HIF-1 regulates CD47 expression in breast cancer cells to promote evasion of phagocytosis and maintenance of cancer stem cells. Proc Natl Acad Sci U S A.

[75] Michaels AD, Newhook TE, Adair SJ (2018). CD47 blockade as an adjuvant immunotherapy for Resectable pancreatic Cancer. Clin Cancer Res.

[76] Liu X, Pu Y, Cron K (2015). CD47 blockade triggers T cell-mediated destruction of immunogenic tumors. Nat Med.

[77] Palazón A, Martínez-Forero I, Teijeira A (2012). The HIF-1α hypoxia response in tumor-infiltrating T lymphocytes induces functional CD137 (4-1BB) for immunotherapy. Cancer Discov.

[78] Lee P, Chandel NS, Simon MC (2020). Cellular adaptation to hypoxia through hypoxia inducible factors and beyond. Nat Rev Mol Cell Biol.

[79] Noman MZ, Buart S, Van Pelt J (2009). The cooperative induction of hypoxia-inducible factor-1 alpha and STAT3 during hypoxia induced an impairment of tumor susceptibility to CTL-mediated cell lysis. J Immunol (Baltimore, Md : 1950).

[80] Noman MZ, Janji B, Kaminska B (2011). Blocking hypoxia-induced autophagy in tumors restores cytotoxic T-cell activity and promotes regression. Cancer Res.

[81] Hasmim M, Noman MZ, Lauriol J (2011). Hypoxia-dependent inhibition of tumor cell susceptibility to CTL-mediated lysis involves NANOG induction in target cells. J Immunol (Baltimore, Md : 1950).

[82] Hasmim M, Janji B, Khaled M (2017). Cutting Edge: NANOG Activates Autophagy under Hypoxic Stress by Binding to BNIP3L Promoter. J Immunol (Baltimore, Md : 1950).

[83] Viry E, Baginska J, Berchem G (2014). Autophagic degradation of GZMB/granzyme B: a new mechanism of hypoxic tumor cell escape from natural killer cell-mediated lysis. Autophagy..

[84] Messai Y, Noman MZ, Hasmim M (2014). ITPR1 protects renal cancer cells against natural killer cells by inducing autophagy. Cancer Res.

[85] Messai Y, Noman MZ, Janji B, et al. The autophagy sensor ITPR1 protects renal carcinoma cells from NK-mediated killing. Autophagy. 2015:0. 10.1080/15548627.2015.1017194 25714778

[86] Terry S, Faouzi Zaarour R, Hassan Venkatesh G (2018). Role of hypoxic stress in regulating tumor immunogenicity, resistance and plasticity. Int J Mol Sci.

[87] Jayaprakash P, Ai M, Liu A (2018). Targeted hypoxia reduction restores T cell infiltration and sensitizes prostate cancer to immunotherapy. J Clin Invest.

[88] Hendricksen K, Cornel EB, de Reijke TM (2012). Phase 2 study of adjuvant intravesical instillations of apaziquone for high risk nonmuscle invasive bladder cancer. J Urol.

[89] Sun X, Kanwar JR, Leung E (2001). Gene transfer of antisense hypoxia inducible factor-1 alpha enhances the therapeutic efficacy of cancer immunotherapy. Gene Ther.

[90] Kheshtchin N, Arab S, Ajami M (2016). Inhibition of HIF-1α enhances anti-tumor effects of dendritic cell-based vaccination in a mouse model of breast cancer. Cancer Immunol Immunother.

[91] Lazarus D, Peters C, Stockmann A, et al. Abstract 3209: CRLX101, an investigational nanoparticle-drug conjugate of camptothecin, demonstrates synergy with immunotherapy agents in preclinical models. 2016;76(14 Supplement):3209-.

[92] Chang DK, Moniz RJ, Xu Z (2015). Human anti-CAIX antibodies mediate immune cell inhibition of renal cell carcinoma in vitro and in a humanized mouse model in vivo. Mol Cancer.

[93] Chafe SC, McDonald PC, Saberi S (2019). Targeting hypoxia-induced carbonic anhydrase IX enhances immune-checkpoint blockade locally and systemically. Cancer immunology research..

[94] Ma SR, Deng WW, Liu JF (2017). Blockade of adenosine A2A receptor enhances CD8(+) T cells response and decreases regulatory T cells in head and neck squamous cell carcinoma. Mol Cancer.

[95] Hatfield SM, Kjaergaard J, Lukashev D (2015). Immunological mechanisms of the antitumor effects of supplemental oxygenation. Sci Transl Med.

[96] Scharping NE, Menk AV, Whetstone RD (2017). Efficacy of PD-1 blockade is potentiated by metformin-induced reduction of tumor hypoxia. Cancer Immunol Res.

[97] Munn LL, Jain RK (2019). Vascular regulation of antitumor immunity. Science (New York, NY).

[98] Llovet JM, Kudo M, Cheng AL, et al. Lenvatinib (len) plus pembrolizumab (pembro) for the first-line treatment of patients (pts) with advanced hepatocellular carcinoma (HCC): Phase 3 LEAP-002 study. 2019;37(15_suppl):TPS4152-TPS.

[99] Hunter FW, Wouters BG, Wilson WR (2016). Hypoxia-activated prodrugs: paths forward in the era of personalised medicine. Br J Cancer.

[100] Phillips RM, Hendriks HR, Peters GJ (2013). EO9 (Apaziquone): from the clinic to the laboratory and back again. Br J Pharmacol.

[101] Bhattarai D, Xu X, Lee K (2018). Hypoxia-inducible factor-1 (HIF-1) inhibitors from the last decade (2007 to 2016): a "structure-activity relationship" perspective. Med Res Rev.

[102] Parayath N, Padmakumar S, Nair SV, et al. Strategies for targeting cancer immunotherapy through modulation of the tumor microenvironment. Regen Eng Transl Med. 2020;6(1):29–49.

[103] Feng LL, Cai YQ, Zhu MC (2020). The yin and yang functions of extracellular ATP and adenosine in tumor immunity. Cancer Cell Int.

[104] Leone RD, Horton MR, Powell JD (2015). Something in the air: hyperoxic conditioning of the tumor microenvironment for enhanced immunotherapy. Cancer Cell.

[105] Haibe Y, Kreidieh M, El Hajj H (2020). Resistance mechanisms to anti-angiogenic therapies in Cancer. Front Oncol.

[106] Hodi FS, Lawrence D, Lezcano C (2014). Bevacizumab plus ipilimumab in patients with metastatic melanoma. Cancer Immunol Res.

[107] Wallin JJ, Bendell JC, Funke R (2016). Atezolizumab in combination with bevacizumab enhances antigen-specific T-cell migration in metastatic renal cell carcinoma. Nat Commun.

[108] Goel S, Duda DG, Xu L (2011). Normalization of the vasculature for treatment of cancer and other diseases. Physiol Rev.

[109] Liapis E, Klemm U, Karlas A, et al. Resolution of spatial and temporal heterogeneity in bevacizumab-treated breast tumors by eigenspectra multispectral optoacoustic tomography. Cancer Res. 2020. 10.1158/0008-5472.CAN-20-1011 32994204

[110] Conley SJ, Gheordunescu E, Kakarala P (2012). Antiangiogenic agents increase breast cancer stem cells via the generation of tumor hypoxia. Proc Natl Acad Sci U S A.

[111] Fukumura D, Kloepper J, Amoozgar Z (2018). Enhancing cancer immunotherapy using antiangiogenics: opportunities and challenges. Nat Rev Clin Oncol.

